# Order of the British Empire

**Published:** 1919-01-18

**Authors:** 


### HONOURS CONFERRED FOR HOSPITAL WORK.

### Order of the British Empire.

Ladies and gentlemen who have distinguished themselves during the war by their untiring efforts on behalf of the sick and wounded are well represented in the formidable list of promotions in and appointments to the Most Excellent Order of the British Empire, which is published in a Supplement to the "London Gazette." Among the ladies honoured will be found the names of Princess Beatrice and Princess Marie Louise, who become Dames Grand Cross. We should like to refer in detail to the services rendered by many whose names appear in the list, but, after all, everyone mentioned has done well in the nation's hour of need, and a simple record of the recipients of honours who are connected with hospital work will perhaps best meet the case. It is as follows :—

Dames Grand Cross (Civil Division).—H. R. H. Princess Beatrice, President of the Isle of Wight Branch, British Red Cross; H.H. Princess Marie Louise, Head of the Bermondsey Voluntary Hosp.; Duchess of Bedford, member of Joint War Committee, British Red Cross Society and Order of St. John; Viscountess Buxton; Miss Sarah Ann Swift, R.R.C., Matron-in-Chief, British Red Cross Society and Order of St. John; Marchioness of Waterford, Head of the Irish War Hospitals Supply Depôts, member of the Joint War Committee for Leinster, Munster, and Connaught, British Red Cross Society and Order of St. John.

Knights Commanders (Civil Division).—Edward Napier Burnett, Esq., J.P., M.D., F.R.C.S., F.R.C.P., Chairman of the Economic Committee of the Army Medical Department, War Office; Sir Frederick Green, League of Mercy; Col. W. Hale White, M.D., R.A.M.C., Chairman and Consultant, Queen Mary's Royal Naval Hosp., Southend.

Dames Commanders.—Miss Rachel Eleanor Crowdy, R.R.C., Principal Commandant, V.A.D.s in France; Lady Blanche Gordon Lennox, Director of H.R.H. Princess Victoria's Rest Clubs for Nurses.

Commanders (Civil Divisions).—Bacon, Mrs. Constance Alice, Deputy President, Norfolk Branch, British Red Cross Society; Arthur Baker, Esq., late British Red Cross Commissioner, Rumania; Lt.-Col. A. J. Barry, T.D., Commandant, Red Cross Society Convoys with the French Commission; Col. William George Beyts, Army Medical Service, Assistant Director of Medical Services, Bombay Brigade; Carnegy, Lady Oliver Beryl, R.R.C., Head of the Naval and Military V.A.D. Department, British Red Cross Society; Lady Gwendoline Colvin, Chairman Executive Committee, Essex Branch, British Red Cross Society; Dale, Henry Hallett, Esq., M.D., F.R.S.; Eichholz, Alfred, Esq., M.D., Senior Assistant Medical Officer, Board of Education; Mrs. Marjorie Robertson-Eustace, Organiser and Administrator of the First Rest Club for Nursing Sisters in France; Fedden, Mrs. Katharine, Chairman of Belgravia War Hospital Supply Depôt; Gilbert, Mrs. Grace Davies, Deputy President, Eastbourne Division, Sussex Branch, British Red Cross Society; the Hon. Mrs. Maud Ernestine Gladstone; Vice-President, Chester City Division, Cheshire Branch, British Red Cross Society; Countess Grey, President, Northumberland Branch, British Red Cross Society; Hastings, Frank, Esq., Secretary, Headquarters Staff, British Red Cross Society· Lyle, Samuel, Esq., M.B., Ch.B., Commissioner of Medical Services, Ministry of National Service; McMordie, Mrs. Julia, M.B.E., President of St. John V.A.D.s for Belfast: Nichols, Charles Lee, Esq., Honorary Auditor, British Red Cross Society and Order of St. John; Obre, Henry, Esq., Chairman of No. 8 Red Cross (Baltic and Corn Exchange) Hospital, Etaples; Pelham, Admiral, County Director, Auxiliary Hospital and V.A.D.s, Sussex; Countess of Pembroke and Montgomery, O.B.E., Vice-President, Wiltshire Branch, British Red Cross Society; Organiser, Wilton House Auxiliary Hospital, Salisbury; Roberts, George Quinlan, Esq., Secretary, St. Thomas's Hospital; Mrs. Florence Haynes-Rudge, Commandant and Donor, Abbey Manor Auxiliary Hospital, Evesham; Thomson, Colonel William Gordon, V.D., D.L., J.P., Red Cross Commissioner, Central Eastern District of Scotland, and Honorary Secretary and Acting County Director for the County of the City of Dundee, Scottish Branch, British Red Cross Society; Lady Mary Katherine Turnor, President, North Lincolnshire Branch, British Red Cross Society; Warre, Capt. George Francis, late Head of Motor Boat Department, British Red Cross Society; Donor of Rest House for Nurses, Rocquebrune, Riviera.

Officers (Civil Division).—Barber, Miss Mabel Emily, Lady Superintendent, H.R.H. Princess Victoria's Rest Club, St. Omer; Richard Dawson Bates, Esq., Honorary Secretary, Craigavon Hospital for Neurasthenics; Miss Agneta Frances Beauchamp, Manager, Red Cross Refreshment Station, Salonika; Mrs. Ethel Bergen, Commandant and Donor of Auxiliary Hospital, Attingham, Shrewsbury; Harold William Meares Binney, Esq., Chief Commissariat Officer and Deputy Director, Middlesex Branch, Joint V.A. Organisation, British Red Cross and Order of St. John; Mrs. Pearl Hall Bishop, Organiser and Commandant, Oakdene Auxiliary Hospital and Annexe, Railhill; Lady Bradford, Lady Helper, Surgical Divisions, Nos. 24 and 26 General Hospitals, British Expeditionary Force; the Hon. Mrs. Maude Helena Brassey, Vice-President, Oxfordshire Branch of the British Red Cross Society, and Donor and Commandant of the Chipping Norton Auxiliary Hospital; the Hon. Mrs. Diana Isabel Brougham, Staff Commandant and Assistant Head of Military Department, Joint Women's V.A.D. Department, British Red Cross and Order of St. John; Mrs. Marion Bruce, Registrar and Organiser of Timberhurst Auxiliary Hospital, Bury; Miss Christiana Mary Burchardt, County Secretary, Oxfordshire Branch, British Red Cross Society; Miss Frances Mary Buxton, Wounded and Missing Inquiry Department, British Red Cross Society; Miss Margaret Byron, Commandant of the Women's Detachment of the London Ambulance Column, City of London Branch, British Red Cross Society; Campion, Miss Mary Gertrude, Commandant of V.A.D.s, Rouen Area, France; Lady Mabel Barclay Cantlie, Red Cross Organiser, Division of Marylebone, County of London Branch, British Red Cross Society; Miss Edith Maud Cliff, Commandant, Gledhow Hall Auxiliary Hospital, Leeds; Blair Onslow Cochrane, Esq., County Director, Auxiliary Hospitals and V.A.D.s, Isle of Wight; Cuthbert Crowley, Esq., Secretary, Kitchener House Club for Wounded Soldiers and Sailors, British Red Cross Society; Mrs. Zella Culley, Vice-President, Alnwick Division, Northumberland Branch, British Red Cross Society; Miss Mabel Jane Cutbush, Organiser and Commandant, Rosslyn Auxiliary Hospital, Hampstead Davis, Mrs. Florence Mary, Nurses' Department, British Red Cross Society; Mrs. Marion Disraeli, Vice-President of the Wycombe and Desborough Division, Buckinghamshire Branch, British Red Cross Society; Mrs. Nora Kathleen Durler, Joint Commandant, Wardown Auxiliary Hospital, Luton; Elliot, Alexander Macbeth, Esq., M.D., Headquarters Medical Examiner, British Red Cross Society; Major-General Philip Mackay Ellis, County Director, Auxiliary Hospitals and V.A.D.s, Carnarvonshire: Mrs. Minnie Enness, Red Cross Work, Egypt; Ferguson, Alfred Cornwall, Esq., M.D., Commandant and Medical Officer, Thirsk Auxiliary Hospital; David John Finlayson, Esq., M.B.E., Chief Accountant, British Red Cross Society; Mrs. Margaret Mary Maitland Fowler, Vice-President, Atherstone Division, Warwickshire Branch, British Red Cross Society; Commandant, Weddington Hall Auxiliary Hospital; Mrs. Florence Fraser, Commandant, Massandra Auxiliary Hospital, Weymouth; Greer, Mrs.

**Honours Conferred for Hospital Work**—*(continued)*.

Olivia Mary, Organiser of Red Cross Work Rooms, Dublin; late Commandant of Lady Dudley's Hospital for Officers, Bournemouth; John Temperley Grey, Esq., M.R.C.S., L.R.C.P., Donor and Medical Officer, Stanmore House Auxiliary Hospital, Lenham; HARDING, John Rudge, Esq., Secretary, Auxiliary Home Hospitals Department, British Red Cross Society; Mrs. Mary Elizabeth Hedley, Vice-President, North Riding of Yorkshire Branch, British Red Cross Society; Administrator and Donor of Hospital at Middlesbrough; Mrs. Eveleen Mary Henderson, Vice-President of Leatherhead Division, Surrey Branch, British Red Cross Society; Commandant of Red House Auxiliary Hospital, Surrey; Mrs. Alice Helen Henry, Organiser and Head of Sphagnum Moss Department, Irish War Hospital Supply Depôt, British Red Cross Society; Mrs. Mary Hiley, Commandant, Sleaford Auxiliary Hospital; Miss Agnes Maud Hulme, Lady Superintendent, H.R.H. Princess Victoria's Rest Club, Calais; Mrs. Katharine Anne Hyde, Donor and Commandant, Wharmton Towers Auxiliary Hospital, Greenfield, Yorkshire; ISAACS, Mrs. Mariette, Commandant of Queen's Gate Auxiliary Hospital, London; JOHNSTONE, Robert William, Esq., M.D., F.R.C.S., Commissioner of Medical Services, Ministry of National Service; KAY, Mrs. Mary Lees, Vice-President of the Middlewich Division, Cheshire Branch, British Red Cross Society; Administrator, Brunner Mond Club Hospital, Middlewich; Miss Alice Cicell King, Commandant, New Court Auxiliary Hospital, Cheltenham; LAURIE, Mrs. Annie Macpherson, Vice-President, Weymouth Division, Dorsetshire Branch, British Red Cross Society; Col. Charles Linton, County Director, Auxiliary Hospitals and V.A.D.s, Huntingdonshire; Lieut.-Col. Llewellyn Wood Longstaff, Vice-President and Assistant County Director, Wimbledon Division, Surrey Branch, British Red Cross Society (to date from November 1, 1918); Mrs. Mary Sophia Loudon, Joint Commandant, Christchurch Auxiliary Hospital, Hampshire; John Reuben Lunn, Esq., F.R.C.S., Commandant, "The Cecils" Auxiliary Hospital, Chappell Croft, Sussex; Miss Mary Fownes Luttrell, Vice-President, Williton District, Somersetshire Branch, British Red Cross Society, Organiser of Red Cross Hospital, Minehead, Somersetshire; MCGRIGOR, Mrs. Magarret Annie Kay, Vice-President, Stirlingshire Branch, Scottish Branch, British Red Cross Society; Miss Elsie Trant MacSwinney, Staff Commandant, Motor and Training Departments, Joint Women's V.A.D. Department, British Red Cross and Order of St. John; Dudley Christopher Maynard, Esq., Architect of the Queen's Auxiliary Hospital, Frognal, Foot's Cray, Kent; Mrs. Ellen Cameron Miller, Red Cross Work, Egypt; Mrs. Irene Helen Miller, Vice-President, Rugby Division, British Red Cross Society; Baroness Mary Florence Edith Mostyn, Commandant and Donor, Mostyn Convalescent Home, Holywell, Flint; Mrs. Catherine Miller-Mundy, Vice-President Ilkeston Division, Derbyshire Branch, British Red Cross Society; NORBURY, Captain Lionel Edward Close, M.B., F.R.C.S., Surgeon, British Red Cross Hospital, Netley; Mrs. May Nuttall, Vice-President, Oldham Division, British Red Cross Society; O'BRIEN, Miss Beatrice Jane, Head of the Sphagnum Moss Branch, Irish Women's Hospital Supply Depôt; PAUL, William Francis, Esq., J.P., Donor and Organiser, Broadwater Auxiliary Hospital; the Hon. Mrs. Mary Olivia De La Poer, Organiser, Tipperary War Hospital Supply Depôt, British Red Cross Society; Indy Beatrice Adine Pretyman, Donor, Orwell Park Auxiliary Hospital; RAIKES, Ernest Barkley, Esq., County Secretary, Norfolk Branch, British Red Cross Society; Major David Valentine Rees, T.D., L.R.C.P., M.R.C.S., Operating Surgeon, Brecon and Builth Auxiliary Hospitals; Charles Reid, Esq., M.B., C.M., Deputy County Director, Staffordshire Branch, British Red Cross Society: Miss Katherine Alice Ricardo, Joint Commandant, Christchurch Auxiliary Hospital; Edward Coleridge Roberts, Esq., M.R.C.S., J.P., Senior Medical Officer, Grovelands Auxiliary Hospital, Southgate, Middlesex; Miss Katharine Haigh Robinson, Commandant, University Auxiliary Hospital, Oxford; John Henry Rogers, Esq., Hon. Secretary and Treasurer, Birmingham Joint V.A.D. Committee, British Red Cross Society; Miss Bessie Rose Row, General Service Superintendent, Devonshire Branch, British Red Cross Society; Mrs. Clementina Elizabeth Hope Rowcliffe, Vice-President, Cranleigh Division, Surrey Branch, British Red Cross Society, Commandant of Oaklands Auxiliary Hospital; Dowager Duchess of Roxburghe, President, Haddingtonshire Branch, British Red Cross Society; the Hon. Eustace Scott Hamilton-Russell, Commandant, Brancepeth Castle Auxiliary Hospital, Durham; SAFFORD, Miss Stella Fanny, Lady Superintendent, H.R.H. Princess Victoria's Rest Club, Camiers; Major Charles Sempill de Segundo, V.D., M.B., B.S., Deputy Commissioner of Medical Services, Ministry of National Service; Prideaux George Selby, Esq., M.R.C.S., L.R.C.P., Medical Officer, Auxiliary Hospital, Sittingbourne; Miss Frances Mary Smith, Commandant, Billingborough Auxiliary Hospital; Miss Sarah Helen Smith, Commandant, Priory Auxiliary Hospital, Cheltenham; George Robert Fabris Stilwell, Esq., M.B., M.R.C.S., Medical Officer, Balgowan Auxiliary Hospital, Beckenham; TUBBS, Mrs. Lucinda, Commandant, Albion House Auxiliary Hospital, Newbury; VAUGHAN, Miss Janet Feliza, Vice-President, Taunton District, British Red Cross Society and Organiser of Priory Schools Auxiliary Hospital, Taunton; Miss Caroline Emily Venables, Commandant, Highland Moors Auxiliary Hospital, Llandrindod Wells; WATSON, Mrs. Dorothy Bannerman, District Secretary, British Red Cross Society, and Commandant, Clifford Street Auxiliary Hospital, York; Mrs. Edith Deverell Watson, Lady Superintendent, H.R.H. Princess Victoria's Club for Nurses, Wimereux; Mrs. Florence Ffaith Hastings-Wheler, Donor and Commandant, Ledston Hall Auxiliary Hospital, Castleford; Mrs. Edith Wilmot, Assistant County Director, Devonshire Branch, British Red Cross Society; Mrs. Helena Jane Wilson, Commandant, Chippenham Auxiliary Hospital; Captain Henry Adrian Fitzroy Wilson, Secretary and Registrar, British Red Cross Hospital, Netley; George Mitchell Winter, Esq., J.P., M.R.C.S., L.R.C.P., Chairman, Torquay Food Control Committee; Mrs. Evelyn Emma Amelia Wren, late Vice-President, Lexden-Winstree Division, Essex Branch, British Red Cross Society.

In addition to the above list, British hospitals at home and abroad have contributed a large number of Members to the Civil Division of the Order.

